# The role of autophagy in viral infections

**DOI:** 10.1186/s12929-023-00899-2

**Published:** 2023-01-18

**Authors:** Tong Chen, Shaoyu Tu, Ling Ding, Meilin Jin, Huanchun Chen, Hongbo Zhou

**Affiliations:** 1 State Key Laboratory of Agricultural Microbiology, College of Veterinary Medicine, Huazhong Agricultural University, Wuhan, 430030 China; 2 Key Laboratory of Preventive Veterinary Medicine in Hubei Province, The Cooperative Innovation Center for Sustainable Pig Production, Wuhan, 430030 China

**Keywords:** Autophagy degradation, Immune response, Selective autophagy, Viral infection, Viral replication

## Abstract

Autophagy is an evolutionarily conserved catabolic cellular process that exerts antiviral functions during a viral invasion. However, co-evolution and co-adaptation between viruses and autophagy have armed viruses with multiple strategies to subvert the autophagic machinery and counteract cellular antiviral responses. Specifically, the host cell quickly initiates the autophagy to degrade virus particles or virus components upon a viral infection, while cooperating with anti-viral interferon response to inhibit the virus replication. Degraded virus-derived antigens can be presented to T lymphocytes to orchestrate the adaptive immune response. Nevertheless, some viruses have evolved the ability to inhibit autophagy in order to evade degradation and immune responses. Others induce autophagy, but then hijack autophagosomes as a replication site, or hijack the secretion autophagy pathway to promote maturation and egress of virus particles, thereby increasing replication and transmission efficiency. Interestingly, different viruses have unique strategies to counteract different types of selective autophagy, such as exploiting autophagy to regulate organelle degradation, metabolic processes, and immune responses. In short, this review focuses on the interaction between autophagy and viruses, explaining how autophagy serves multiple roles in viral infection, with either proviral or antiviral functions.

## Highlights

1. This review focuses on the interaction between autophagy and viruses, explaining how autophagy serves multiple roles in viral infection, with either proviral or antiviral functions.
2. Based on different steps of autophagy and the regulation of immune responses by autophagy, this review oversees the role of autophagy in viral replication, maturation, egress and cell–cell spreading.
3. This review provides an important foundation for the development of broad-spectrum antiviral treatment strategies and drugs based on the regulation of autophagy.

## Background

Autophagy is an evolutionarily conserved catabolic process of protein and organelle degradation that is essential to maintain cellular homeostasis [1]. Depending on mechanisms for delivery of cargo to lysosomes, autophagy is categorized into the microautophagy and macroautophagy and chaperone-mediated autophagy (CMA). In microautophagy, autophagic cargo is directly sorted into lysosomes. In the CMA, before substrate delivery to lysosomes, recognition sites on the autophagic cargo are required for molecular chaperons binding to form the CMA substrate/chaperon complex. Macroautophagy, which will be discussed in-depth in this review (hereafter referred to as autophagy), involves the formation of autophagosome, the major lysosomal pathway for cytoplasmic components turnover.

Viruses are microbes with simple structures that must parasitize living cells to proliferate. Nevertheless, viruses have far greater diversity than other organisms. By 2019, 14 orders, 143 families, 64 subfamilies, 846 genera and 4,958 species of viruses had been discovered according to the International Committee on Taxonomy of Viruses (ICTV; https://talk.ictvonline.org/) [2]. In general, the life cycle of most viruses comprises several distinct stages: (1) attachment/adsorption, (2) entry, (3) uncoating, (4) mRNA production/transcription, (5) synthesis of viral components, (6) assembly, and (7) release.

As a defense strategy of organisms, autophagy can be triggered to antagonize viral infections by delivering cytoplasmic virions or viral components to lysosomes for degradation. In addition, the degradation also promotes the inflammatory response, antigen presentation, and clearance for pathogen recognition [3]. However, previous studies have shown that some viruses inhibit or evade autophagy, whereas some viruses even hijack the autophagy mechanism or exploit autophagy to circumvent the host immunity mechanisms for their benefit. With the onset of the global COVID-19 epidemic, the relationship between autophagy and viruses has attracted increased scientific attention. Some new discoveries have broadened our understanding of the relationship between autophagy and viruses. Notably, virus-specific induction of autophagy is related to endosomes. A virus can trigger the autophagy-related 8-phosphatidylserine (ATG8-PS) alternative lipidation mechanism, as well as several others, but this remains poorly understood. Based on different steps of autophagy and the regulation of immune responses by autophagy, this review oversees the role of autophagy in viral replication, maturation, egress and cell–cell spreading.

## Autophagy

### The process and regulation of autophagy

More than 30 ATGs reported to date participate in the following four steps of autophagy:

1. Autophagy initiation

**Fig. 1:**
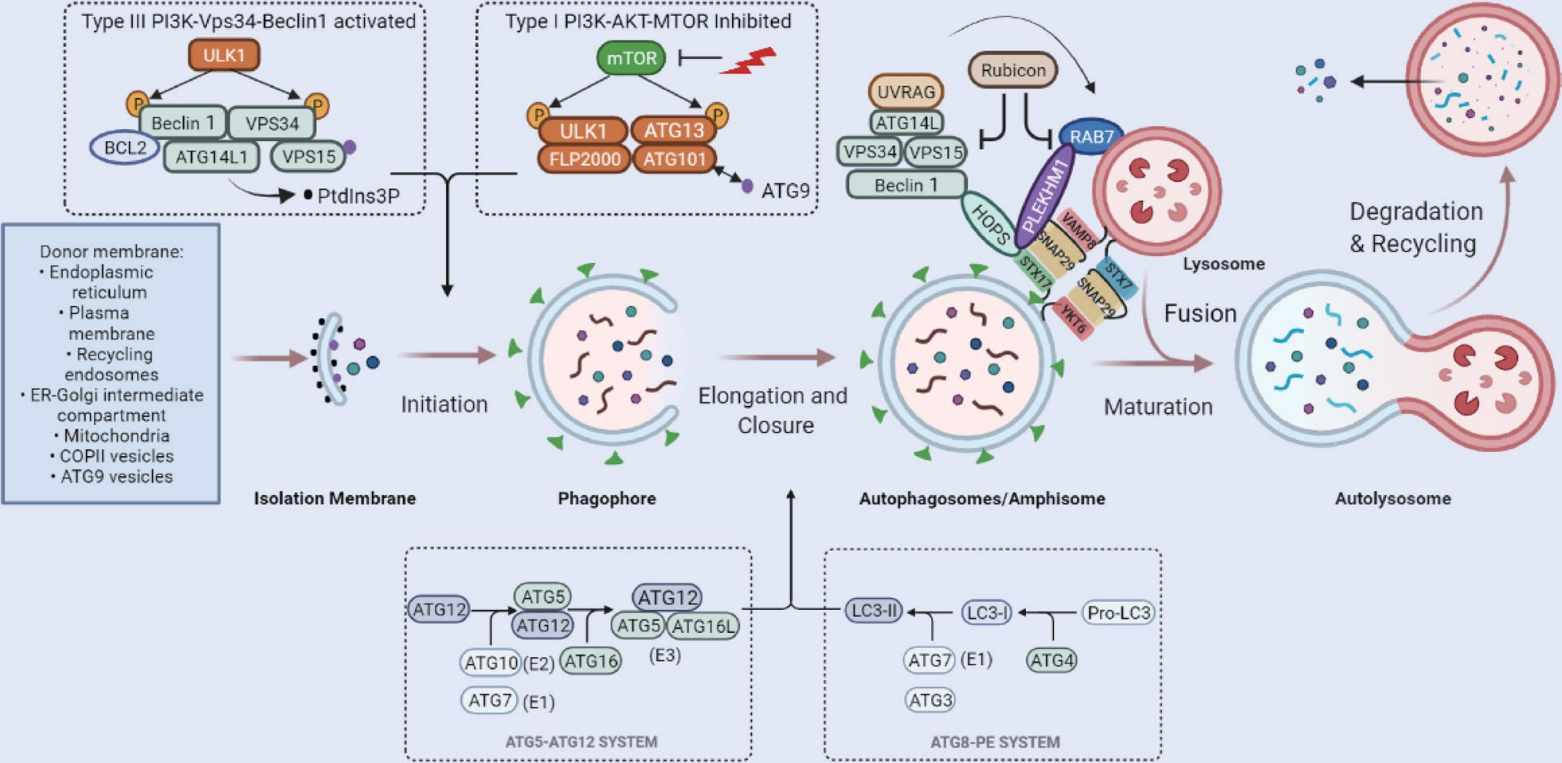
The process and regulation of autophagy. Autophagosomes are derived from IM produced on various organelles. Under stress conditions, the type III PI3K-Vps34-Beclin1 complex is activated, and type I PI3K-AKT-MTOR signalling pathway is inhibited. mTOR inhibition allows ULK1 and FIP2000 to re-associate with dephosphorylated ATG13 and also causes mATG9 to redistribute from TGN to the late endosome, thus forming an IM; it also dephosphorylates and activates the ULK1-ATG13-FIP200-Atg101 complex, leading to autophagy initiation. In parallel, the Beclin1-ATG14L-Vps15-Vps34 complex is activated to generate PtdIns3P on the endomembrane. Elongation and closure of the autophagic membrane require two ubiquitin-like conjugation systems. ATG12 is covalently conjugated to ATG5 with the assistance of ATG7 (encodes an E1-like enzyme) and ATG10 (encodes an E2-like enzyme), then binds with ATG16 and multimerizes to form the ATG12-ATG5-ATG16L complex, which forms an E3-like ligase of LC3, which oligomers coat on the surface or tips of phagophore to initiate its elongation and curvature. The second is the ATG8-PE system: The PE is conjugated to pro-LC3 under the continuous action of ATG4, ATG7 and ATG3 to form LC3-II, respectively. LC3-II incorporates itself into the autophagosome membrane to drive the extension and closure. The maturation of autophagosome is mediated by SNARE, Rab GTPase family members, and Tethering factors. Two cognate SNARE complexes, STX17-SNAP29-VAMP8 and YKT6-SNAP29-STX7, mediate autophagosome and lysosome fusion. Tethering factors, such as HOPS trap vesicles, bring the SNARE complex closer to the target membrane during their intracellular transport. HOPS components promote autophagosome-lysosome fusion through interaction with STX17. In addition, Rubicon negatively regulates the endosome or autophagosome maturation through VPS34, ATG14L or interactions with Rab7 and UVRAG, but Rab7 facilitates the binding of the autophagosome to the HOPS complex on the lysosomes through PLEKHM1. UVRAG activates PI3KC3 and C-VPS/HOPS. Finally, engulfed proteins or organelles are degraded by lysosomal enzymes in autolysosomes, and LC3B-II is also degraded and recycled

In general, autophagosomes are derived from the isolation membrane (IM) produced on various organelles, including the endoplasmic reticulum (ER), plasma membrane, recycling endosomes, mitochondria, ATG9-vesicles, COPII vesicles, and ER-Golgi intermediate compartment (ERGIC) [4]. Under stress, cellular type III PI3K-Vps34-Beclin1 complex is activated, and type I PI3K-AKT-MTOR signalling pathway is inhibited. Mechanistic target of rapamycin (MTOR) inhibition allows ULK1/ATG1 and FIP200/RB1CC1/ATG17 to re-associate with dephosphorylated ATG13, and also causes mATG9 to redistribute from the trans-Golgi network (TGN) to the late endosome and form a cup-shaped double-layer IM, and dephosphorylate and activate the ULK1-ATG13-FIP200-ATG101 complex, leading to the initiation of autophagy [5]. In parallel, the Beclin1-ATG14L-VPS15-VPS34 complex is activated to generate phosphatidylinositol-3-phosphate (PtdIns3P) on the endomembrane [6]. The PtdIns3P-enriched area on the endomembrane surface is termed phagophore, which provides a platform for the IM nucleation and expansion (Fig. 1) [7].

(2) Elongation and closure of the autophagic membrane

Two ubiquitin-like conjugation systems are required in this process. The first is the ATG5-ATG12 ubiquitin-like protein conjugation system: ATG12 is covalently conjugated to ATG5 with the assistance of ATG7 (encodes an E1-like enzyme) and ATG10 (encodes an E2-like enzyme). Then, ATG12-ATG5 complex binds ATG16 and multimerizes to form the ATG12-ATG5-ATG16L complex, which forms an E3-like ligase of the microtubule-associated protein L chain 3 (LC3) [8]. The oligomers of E3-like ligase of LC3 coat the surface or tips of phagophores to initiate their elongation and curvature [8].

The second ubiquitin-like conjugation system is the ATG8-phosphatidylethanolamine (PE) system: the PE is conjugated to Pro-LC3 under the continuous action of ATG4, ATG7 and ATG3, respectively. Specifically, Pro-LC3 is cleaved by ATG4 to produce a soluble form of LC3-I (non-lipidated,18 kDa). LC3-I is activated by ATG7 and transferred to ATG3, and then modified into an autophagy-related form of LC3-II (the combined form of PE, 16 kDa). Moreover, LC3 is present in two forms: LC3-I and LC3-II. In unstimulated cells, LC3 is mainly located in the nucleus, with only a small proportion located in the cytoplasm. When autophagy is activated by external stimuli, pro-LC3 is cleaved into LC3-I and LC3-II. LC3-I dissociates in the cytoplasm into a soluble form, while LC3-II incorporates itself into the autophagosome membrane to drive the extension [9] and closure [10] of the membrane. Thus, the net amount of LC3-II is a critical hallmark for monitoring autophagy (Fig. 1).

(3) Maturation and fusion with the lysosomes of autophagosomes

The autophagosome undergoes maturation (including cargo material packaging), then gets transported to lysosomes through the cytoskeletal structures, and finally fuses with the lysosome, leading to the formation of autolysosomes. This process is mediated by intracellular proteins involved in the vesicle transport and fusion, especially soluble N-ethylmaleimide-sensitive factor attachment protein receptor (SNARE) superfamily members (YKT6, STX17, SNAP29, VAMP3, VAMP7, VAMP8 and VTI1B) [11–15] Rab GTPase family members (RAB7, RAB8B, RAB9, RAB11, RAB23, RAB24 and RAB33) [16–20] and tethering factors (HOPS complex: vacuolar protein sorting 11 (VPS11), Vps16, VPS18, Vps33A, VPS39, and Vps41) [21, 22]. Two cognate SNARE complexes, STX17-SNAP29-VAMP8[13] and YKT6-SNAP29-STX7 [23] function additively in mediating fusion of the autophagosome with lysosome. Tethering factors trap vesicles during their intracellular transportation and bring them closer to the target membrane, thereby further stabilizing the assembly of SNARE to enhance the specificity and efficiency of vesicle fusion [24]. Through synergistic binding with the Rab protein, SNARE and phospholipids, Tethers are recruited to specific membranes [24, 25]. For instance, all HOPS components promote autophagosome-lysosome fusion through interaction with STX17 [26]. In addition, Rubicon negatively regulates the endosome or autophagosome maturation through VPS34, ATG14L or interactions with Rab7 and the ultraviolet radiation resistance-associated gene protein (UVRAG) [27–29]. Rab7 facilitates binding of the autophagosome to the HOPS complex on the lysosome through the pleckstrin homology domain-containing family M member 1 (PLEKHM1) [30]. UVRAG, a component of the PI3KC3 complex (VPS34, p150, Beclin1, UVRAG and ATG14L), functions as a guanine nucleotide exchange factor that catalyzes the exchange of GDP for GTP on Rab7, which activates PI3KC3 and C-VPS/HOPS (Fig. 1) [31].

(4) Autophagosome degradation and recycling

In autolysosomes, engulfed proteins or organelles are eventually degraded by lysosomal enzymes, and LC3B-II is also degraded and recycled (Fig. 1).

### The types of autophagy

**Fig. 2:**
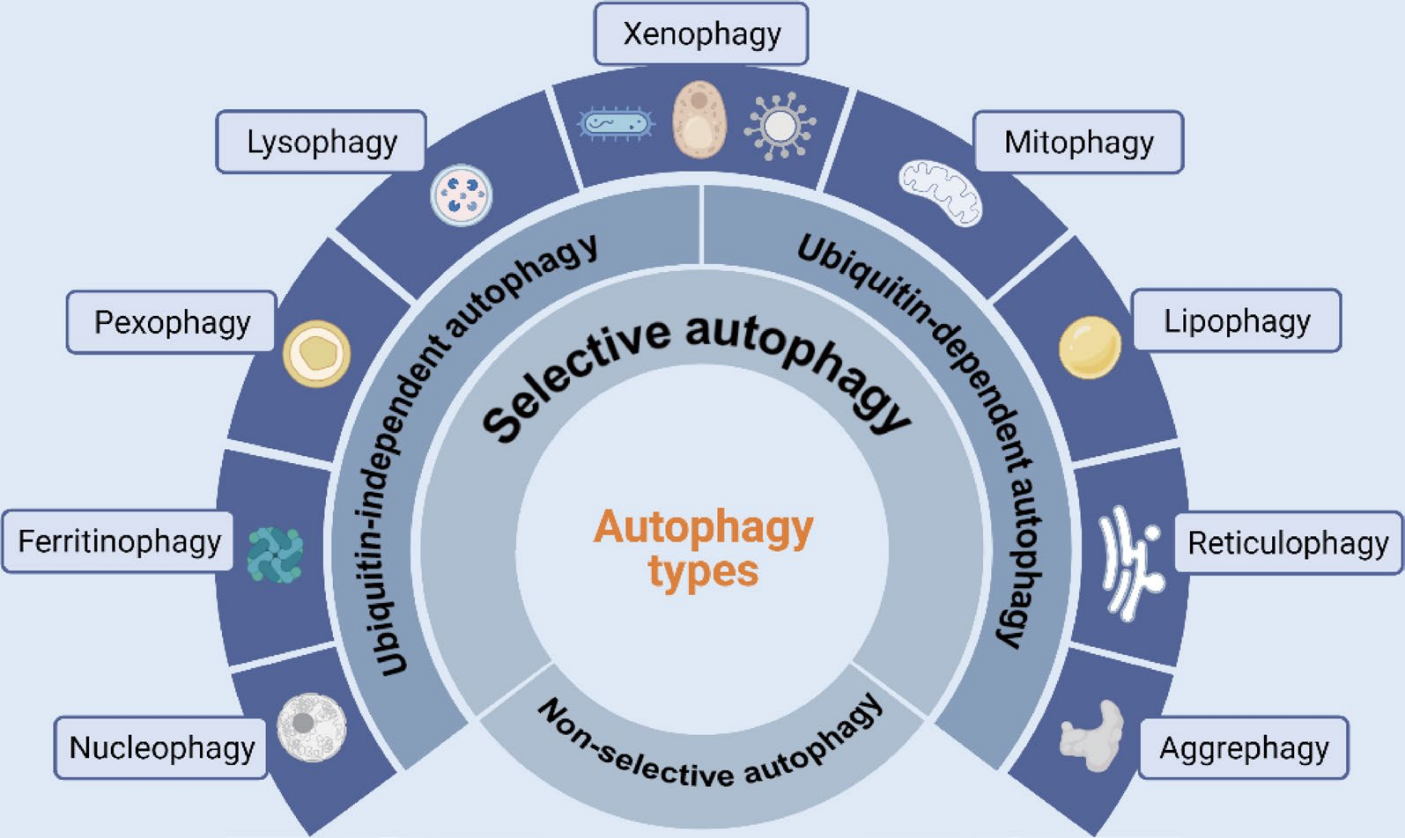
The types of autophagy. autophagy can be divided into selective autophagy and non-selective autophagy according to nutritional status. Selective autophagy, which is mediated by specific receptors, can be further divided into the ubiquitin-dependent and independent autophagy. Furthermore, selective autophagy has been well characterized and classified according to the type of targeted cargo. For instance, nucleophagy (nuclei), ferritinophagy (ferritin), pexophagy (peroxisomes), lysophagy (lysosomes), xenophagy (intracellular pathogens including bacteria, fungi and viruses), mitophagy (mitochondria), lipophagy (lipid droplets), reticulophagy (endoplasmic reticulum), aggrephagy (protein aggregates)

On the basis of nutritional status, autophagy can be roughly divided into selective autophagy, under nutrient-rich conditions, and non-selective autophagy, under starvation conditions [32, 33]. The non-selective autophagy is conserved and mediated by the ULK1/2 complex [34]. Selective autophagy, which is mediated by specific receptors, can be further divided into the ubiquitin-dependent and independent autophagy [35]. Ubiquitin-dependent selective autophagy involves a group of sequestosome-like receptors (SLRs) [36], including p62/SQSTM1 [37], neighbor of BRCA1 gene 1 (NBR1) [38], TAX1BP1 [39], calcium-binding and coiled-coil domain-containing protein 2 (CALCOCO2/NDP52) [40], optineurin, CCDC50 [41] and CCPG1 [42]. Ubiquitin-independent selective autophagy directly targets cargo to ATG8-containing autophagosome membranes; receptors such as BNIP3 [43], PHB2 [44], NIX/BNIP3L [43], FAM134B [45], FUNDC1 [46], TBC1D5 [47], STBD1 [48] and a newly discovered UIM-type autophagy receptor [49] are involved in this process. Furthermore, selective autophagy targets not only pathogenic microorganisms but also specific cellular components and organelles, and has been well characterized and classified according to the type of targeted cargo. For instance, aggrephagy (protein aggregates), ER-phagy or reticulophagy (endoplasmic reticulum), lipophagy (lipid droplets), mitophagy (mitochondria), nucleophagy (nuclei), lysophagy (lysosomes), pexophagy (peroxisomes), ferritinophagy (ferritin), and xenophagy (intracellular pathogens including bacteria, fungi and viruses) (Fig. 2).

As opposed to the canonical autophagy, non-canonical autophagy precedes the formation of autophagosomes [50], which means that lipidated LC3 is inserted into single membranes, especially the endolysosomal membrane, during the process of cellular engulfing of foreign bodies, such as LC3-associated phagocytosis (LAP) [51]. A proportion of the receptor signalling allows cargo to be recruited to the single membrane vesicle, which leads to its labelling with lipidated LC3-PE. Mechanically, non-canonical autophagy may bypass some steps of canonical autophagy during the formation of functional autophagosomes. For instance, it may bypass proteins that are critical for nucleation (Beclin1) and initiation (ULK1), and other proteins involved in elongation and closure (ATG7, ATG5) [52].

There exists another autophagy type: secretory autophagy, which exerts biological functions in the unconventional secretion of leaderless cytosolic proteins [53]. As opposed to proteins that have the N-terminal leader peptides, leaderless cytosolic proteins cannot get into the regular secretory pathway normally operating through the Golgi apparatus and ER [54].

## Viruses manipulate the autophagy process

### Virus-mediated autophagy initiation

#### Viral infection induces autophagy initiation

**Fig. 3:**
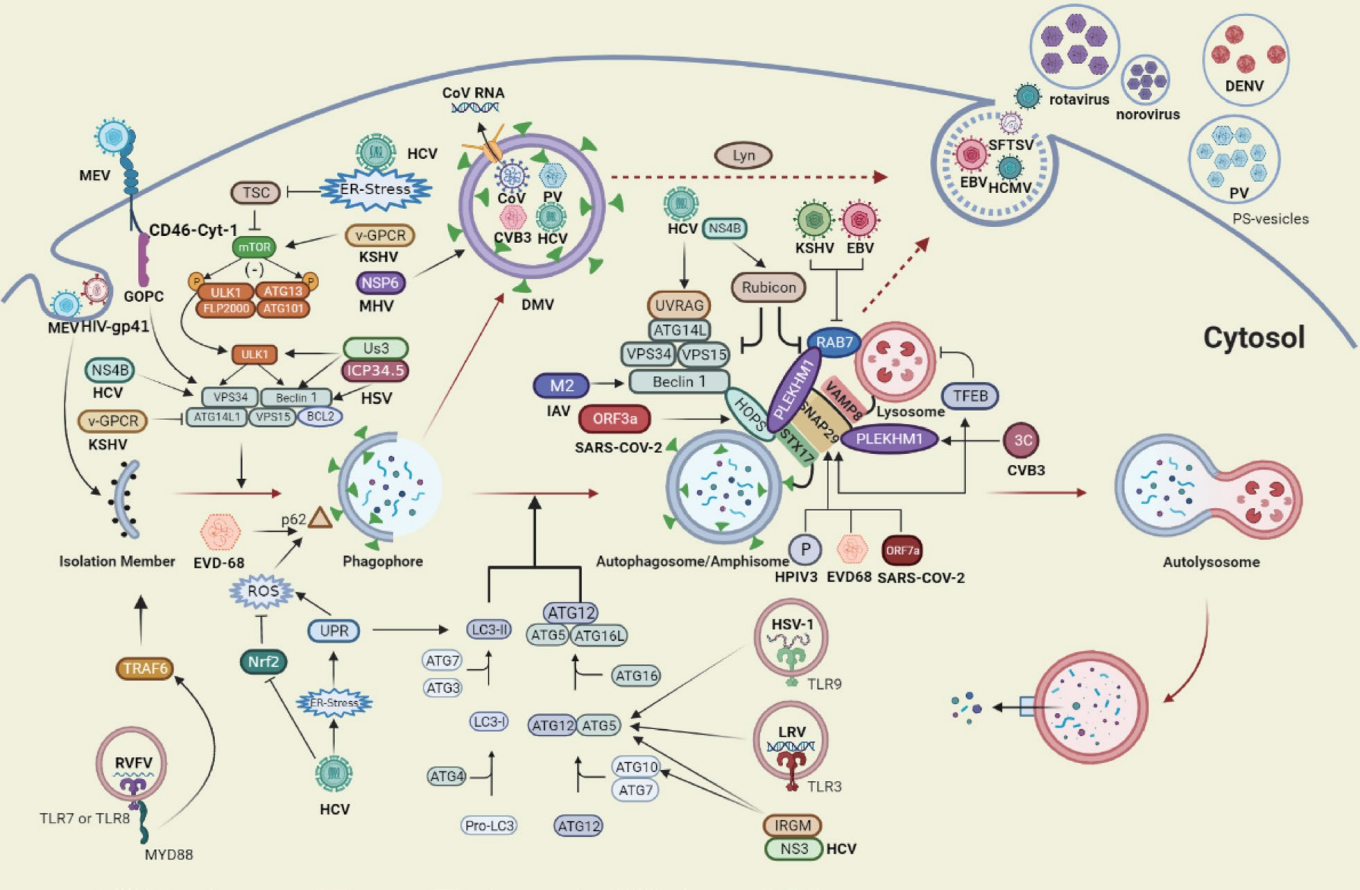
Viruses manipulate the autophagy process. Viruses and viral proteins induce autophagy initiation at different stages of the viral life cycle. In the adsorption stage, MEV combines with CD46-Cyt-1, which is linked to VPS34/Beclin1 complex through the interaction with the GOPC, promoting the formation of autophagosomes. LRV activate TLR3 and TRIF to trigger ATG5-mediated autophagy; ATG5 facilitates the production of TLR9-induced IFN-I in pDCs infected with HSV-1; TLR-7 recognizes RVFV that activates antiviral autophagy through TRAF6 and MyD88. HCV-encoded NS4B triggers the initiation of autophagy by forming a complex with Rab5 and Vps34. Conversely, HSV-1-encoded ICP34.5 binds with Beclin1; v-GPCR encoded by KSHV negatively regulates autophagy. At later stages of autophagy, viruses utilize DMVs as replication or assembly sites. MHV NSP6 induces autophagy to produce DMVs. These DMVs possess double-membrane-spanning molecular pores, which allows RNAs to be exported to the cytosol. CVB3 exploits autophagy to support its replication in DMVs. Virus blocks the fusion of autophagosomes and lysosomes mainly by targeting the SNARE protein, Rab GTPase family and Tethering factors, or disrupting lysosomal function. CVB3 protease 3C, HPIV3 P protein and EVD viral protease target SNAP29 to inhibit autophagy flux. In addition, CVB3 proteinase 3C targets TFEB for proteolytic processing to disrupt lysosomal function. HCV negatively regulates and positively regulates the maturation of autophagosomes by inducing Rubicon or UVRAG, respectively. KSHV and EBV downregulate RAB7 to block autophagy. SARS-COV-2 ORF3a protein sequestrates and interacts with the HOPS component, and ORF7a reduces the fusion with lysosomes. IAV M2 interacting with Beclin1 may prevent the fusion of autophagosomes and lysosomes. Finally, the virus exploits secretory autophagy to promote viral maturation, egress and cell–cell spreading. DENV takes advantage of autophagy-associated vesicles to promote virus transmission. PV is captured by PS lipid-enriched autophagosome-like vesicles, then vesicles are released from cells. EBV or HCMV recruits autophagy-related protein-coupled membranes to its envelope

Any steps of the viral life cycle or exposure to viral proteins may trigger autophagy (Fig. 3). Below, we describe several representative examples to illustrate how viruses induce the initiation of autophagy.

At the stage of virus adsorption, autophagy is usually activated through pathogen receptors, such as CD46. After binding with measles virus (MEV), CD46-Cyt-1 (one of the two C-terminal splice variants of CD46) is linked to the VPS34/Beclin1 complex through interaction with the scaffold protein GOPC, which promotes autophagosome formation [55]. Autophagy is also induced when viruses enter cells through endocytosis and the viral envelope fuses with the endosomal membrane to release its own genetic material. Evidence showed that various members of paramyxoviruses and human immunodeficiency virus (HIV) trigger the formation of autophagic spots through membrane fusion, mainly by envelope glycoproteins [56, 57]. The release of genetic material after fusion activates cytoplasmic pattern recognition receptors (PRR) to induce autophagy, which will be described in detail in section “5. autophagy and innate immunity in virus infection”. Subsequently, perturbation of intracellular environment caused by viral replication in the organelle membranes leads to up-regulated autophagy. ER stress and increased ROS induced by HCV replication also trigger autophagy. ER stress is activated through the accumulation of viral proteins, which trigger the unfolded protein response (UPR) to restore homeostasis. Hepatitis C virus (HCV) infection-induced ER-stress inhibits AKT-tuberous sclerosis complex (TSC), then the TSC inhibits the MTOR pathway to induce autophagy [58]. Simultaneously, the UPR signalling pathway is required for promoting the lipidation of LC3 protein and elevation of ROS in response to the HCV infection through the activation of the ATF6 or IRE1 pathways [59]. Moreover, HCV impairs the activation of Nrf2, leading to elevated ROS levels, which up-regulates the phosphorylation level of p62 [60]. Finally, the newly synthesized viral proteins directly or indirectly target autophagy genes to induce the formation of autophagosomes. For example, the HCV-encoded NS4B is capable of initiating autophagy by forming a complex with Rab5 and VPS34 [61]; and human immunity-related GTPase family M (IRGM) protein interacts with HCV NS3 and autophagy genes (ATG5, ATG10, LC3) to promote the lipidation of LC3, thus promoting the formation of autophagosomes [62].

#### Viral infection suppresses autophagy initiation

Given that autophagy is a part of the antiviral defense mechanism, it is not surprising that viruses evolved mechanisms that allow them to counteract this process. It is mainly achieved by the regulation of viral proteins targeting ATGs, especially for herpesviruses, which are highly adapted to their hosts (Fig. 3).

Herpes simplex virus type 1 (HSV-1)—encoded ICP34.5 was firstly reported to affect autophagy by interacting with Beclin1[63]. Similarly, viral BCL-2 protein and IRS1 and TRS1 encoded by the human cytomegalovirus (HCMV) were also reported to bind with Beclin1, thus impairing the autophagosome formation [64, 65]. A recent study showed that α-herpesvirus Akt-like Ser/Thr kinase limits autophagy in favor of its replication through inhibition of ULK1 and Beclin1 [66]. Subsequently, the v-G protein-coupled receptor (v-GPCR) encoded by Kaposi’s Sarcoma-associated Herpesvirus (KSHV) was reported to negatively regulate autophagy by activating the mTOR pathway; it also mimics the cellular homolog GPCR to down-regulate the ATG14L expression, thus inhibiting autophagy [67, 68]. Therefore, the inhibitory effect of the virus in the initial stage of autophagy can be roughly divided into two categories: the activation of the type I PI3K-AKT-MTOR signalling pathway, or inhibition of the type III PI3K-VPS34-Beclin1 pathway.

### Autophagy hijacked by viruses

At a later stage of autophagy, accumulating evidence suggests that different types of viruses have developed their own unique strategies to inhibit, evade, or manipulate the process of autophagy to achieve the goal of survival and propagation (Fig. 3).

#### Viruses utilize double-membrane vesicles as replication or assembly sites

Coronaviruses (CoV) infection induces autophagy pathway and leads to the formation of DMV for its replication; this comprises viruses such as the mouse hepatitis virus (MHV) [69], Middle East Respiratory Syndrome Coronavirus (MERS-CoV) [70], Severe Acute Respiratory Syndrome Coronavirus (SARS-CoV) [71] and SARS-CoV-2 [72, 73]. Nascent viral RNAs were observed in DMVs within cells infected with MERS-CoV, SARS-CoV [70] and gamma-CoV or SARS-CoV-2 [72, 74] by 2D and 3D analysis of viral replication organelles, indicating that DMVs represents the central hub of viral RNA synthesis. Furthermore, a recent authoritative report identified that these DMVs possesses double-membrane-spanning molecular pores, which allows RNA export to the cytosol [75]. MHV NSP6 activates autophagy flux and induces autophagosome formation from ER, while MHV fails to induce DMVs formation in mouse embryonic stem cells lacking ATG5 [76]. The MHV replication levels in mouse embryonic stem cells lacking the ATG5 were significantly reduced compared with cells expressing the ATG5 [77]. This evidence indicates that the replication of coronaviruses is heavily dependent on autophagy-induced DMVs. However, evidence suggested that LC3 protein exists on DMVs and co-localizes with the MHV replication complexes (p22 and N), but other studies demonstrated that non-structural proteins (nsps) from the RNA replication complex do not colocalize with LC3 [78, 79]. This inconsistency may be caused by LC3: a study showed that endogenous LC3 co-localizes with nsps, while exogenously expressed GFP-LC3 does not [80]. In contrast, despite the autophagy seemingly promoting the replication of coronaviruses, it is not necessary in primary murine embryonic fibroblasts (pMEFs) (it can replicate without the ATG7) [80]. Furthermore, non-lipidized LC3-I covers CoV-induced DMVs, implying an autophagy-independent role for nonlipidated LC3-I [80, 81]. Interestingly, the latest research showed that β-CoV hijacks lysosomes rather than the more commonly biosynthetic secretory pathway exploited by other enveloped viruses, for egress, but this process does not seem to be related to autophagy [82]. The strongest evidence is that fractionation by Nycodenz gradients proves that LC3 is not enriched at the MHV genomic RNA containing-fractions in MHV-infected cells; moreover, a similar assay revealed that LC3 and poliovirus (PV) genomic RNA are enriched at the same fractions [82].

Picornaviruses induce DMVs formation to promote its replication, but the origin of DMVs is yet to be identified. PV was the first found to induce the autophagosome membrane rearrangement [83]. Special DMVs with an autophagy-like structure were observed in PV-infected cells [83–85]. Blocking the formation of autophagosomes inhibits viral RNA synthesis and subsequent steps of the PV life cycle; however, hindering the acidification of vesicles only inhibits the final stage of viral particles maturation [86]. Virion assembly and maturation of PV may occur in various cellular compartments, so the acidic mature autophagosomes may be used as assembly sites. However, there are also studies showing that PV dsRNA does not co-localize with GFP-LC3, implying that its replication may not occur in autophagosomes [87]. Electron microscopy analysis observed several DMVs in HEK293A and Hela cells infected with coxsackievirus B3 (CVB3) [88], and usurpation of autophagosome supports CVB3 replication [88–90]. Nevertheless, autophagy is not absolutely required, Alirezaei et al. reported that the membrane source of DMVs varies and that autophagic membrane may be just one of its origins [91].

Other DMVs derived from cells infected with other viruses such as HCV [92, 93], human norovirus (huNoV) [94] and arterivirus [95] share similar structural characteristics with DMVs originating from a complex ER network. The nsps of these viruses serve critical functions in inducing the DMVs formation. DMVs contain viral nsps, RNA, and enzymatically active replicase in HCV-infected cells. Therefore they are bona fide viral replication organelles sites, but the role of DMVs in the replication of the other two viruses remains to be deciphered.

#### Virus blocks fusion of autophagosomes with lysosomes

There is evidence that picornaviruses target the SNARE protein complex or disrupt lysosomal function to block autophagy degradation. For instance, CVB3 targets SNAP29 and the adaptor protein PLEKHM1, thus inhibiting autophagy flux by impairing the assembly of the SNARE complex through the catalytic activity of viral protease 3C [96]. In another report, the autophagic flux of CVB3-infected cells was restored by overexpressing another component of the SNARE complex, STX17 [97]. In enterovirus 68 (EV-D68)-infected Hela cells, accumulation of GFP-LC3 spot and cleavaged-SNAP29 by viral protease was simultaneously detected [98]. Transcription factor EB (TFEB), which is targeted for proteolytic processing to disrupt lysosomal function and enhance viral infection, has been identified as a new target of CVB3 proteinase 3C [99]. In addition, a recent study found for the first time that incomplete autophagy can be induced during rhinovirus C (RV-C) infection, but the specific mechanism remains to be studied [100].

Similarly, human parainfluenza virus type 3 (HPIV3) is capable of inducing abnormal accumulation of autophagosomes. The P protein of HPIV3 competitively binds to the SNARE regions of SNAP29, and the binding hinders the interaction of SNAP29 and STX17, thus obstructing the fusion of autophagosomes with lysosomes, and increasing the production of extracellular viral particles [101].

Unlike the aforementioned reports, the fusion of autophagosomes with lysosomes is delayed by the regulation of Rubicon [102], UVRAG [102] and UPR [103–105] in different stages of HCV infection. Specifically, at the early stages of HCV infection, NS4B induces Rubicon to inhibit fusion of the autophagosome with lysosomes and promotes the HCV replication; at the late stage of infection, UVRAG is also upregulated and facilitates the maturation of autophagosomes and suppresses HCV replication [102].

Influenza A virus (IAV) infection prevents the late stage of autophagosome maturation. The IAV M2 protein was reported to co-localize with autophagosomes, and plays essential roles in inhibiting the fusion of autophagosomes with lysosomes [106]. Other studies have shown that the interaction between M2 and Beclin1 may prevent the fusion of autophagosomes with lysosomes [28, 106, 107].

The physiological level of autophagy prevents cancer progression by suppressing benign tumour growth, but some oncogenic viruses of the Herpesviridae family induce cancer by dysregulating autophagy, typically exhibiting abnormal accumulation of p62/SQSTM1 [108]. KSHV induces autophagy by replication and transcription activator (RTA), but it downregulates RAB7 to block the final stage of autophagy [109, 110]. Likewise, Epstein-Barr virus (EBV) regulates autophagy through the same strategy to establish stable latent infection [111]. Interestingly, Pringle et al. found that mTORC1 is dispensable for KSHV’s protein synthesis, genome replication, and the release of infectious progeny virions, which means that the virus may have subverted the controlling role of mTOR to autophagy at this stage [112].

Finally, some recent studies showed that SARS-COV-2 possesses a unique strategy to block autophagy. The ORF3a protein sequestrates and interacts with VPS39 to block the fusion of autophagosome/amphisome with lysosomes. Interestingly, ORF3a of SARS-COV does not exert similar capabilities, which may lead to the unique pathogenicity and infectivity of SARS-CoV-2 [113]. Moreover, ORF7a of SARS-CoV-2, another potent autophagy antagonist, reduces the fusion efficiency by down-regulating the acidity of lysosomes [114, 115]. Results from host cells’ network and transcriptome profiling showed that upregulated GSK3B or downregulated SNAP29 may also contribute to mitochondrial and autophagic dysfunctions during the SARS-CoV-2 infection [115, 116].

#### Secretory autophagy promotes viral maturation, egress and cell–cell spreading

The impact of secretory autophagy on virus maturation, egress, and cell–cell spreading has gained increasing interest in recent years. Flaviviruses, including Zika virus (ZIKV), HCV, and West Nile virus (WNV), and dengue virus (DENV), benefit from the autophagy process, and they are heavily dependent on the availability of the ER membrane during their replication [117–121]. Such reliance provides a theoretical framework for secretory autophagy to promote maturation and release of virus particles and cell–cell spreading. The most robust evidence is that the vesicles secreted by DENV-infected cells contain viral proteins E, prM/M, NS1, and viral RNA, as well as the host LC3-I and lipid droplets [122]. These autophagy-associated vesicles not only allow virus transmission but also avoid antibody neutralisation [122]. Meanwhile, inhibition of autophagy deranges the dengue virion maturation [122, 123]. The latest research also showed that Lyn is critical for virus particles enclosing within membranes to secrete; this process depends on SNARE complexes, ULK1, and Rab GTPases, and occurs with much faster kinetics than the conventional secretory pathway [124]. However, the secretory autophagy hijacked by HCV and ZIKV may cross-talk with the exosomal pathway, but this needs further confirmation [125–129].

Enteroviruses, including PV, CVB3 and rhinovirus, hijack the autophagy pathway to spread effectively in the host by being packaged within the vesicles [130–133]. Clusters of PV particles are caught by PS lipid enriched autophagosome-like vesicles and released non-lytically from cells. Importantly, it allows multiple viral RNA molecules to be collectively and efficiently transferred into other cells [130]. In the enteric viral infections, these vesicle-cloaked norovirus and rotavirus clusters remain intact during the fecal–oral transmission between individuals, which allows them to be transferred to the next host [134]. Compared with animals ingesting the same amount of free viruses, this mode of transmission leads to more severe clinical symptoms [134]. In addition, Giansanti et al. recently discovered that inhibition of mTORC1 activates TFEB during enterovirus infection, which up-regulates autophagy and lysosomal genes expression, and that TFEB activation promotes the release of virus particles in extracellular vesicles through secretory autophagy [135]. These strategies enable viruses to spread more effectively in or between hosts and evade the direct effect of antiviral drugs to some extent.

Secretory autophagy is also involved in the maturation and release of bunyavirus and herpesviruses. Autophagy is induced under severe fever with thrombocytopenia syndrome virus (SFTSV) infection, and autophagosome serves as SFTSV assembly platform. SFTSV was also observed to egress from autophagic vacuoles[136]. EBV limits lysosomal degradation of viral components for its own benefits as mentioned above. In the subsequent process, EBV was reported to hijack autophagic vesicles as assembly sites and promote the maturation and export of viral particles [111, 137, 138]. Nowag et al. reported that LC3-II is present in purified virus particles as EBV recruits ATG8/LC3-coupled membranes to its envelope [137]. Electron microscopic analysis showed that autophagic vesicles delivered viral particles to the plasma membrane. In addition, some new studies disclosed that autophagy also interferes with genome replication, morphogenesis, and progeny release of HCMV [139–141]. Results show that not only LC3-II, but also autophagy receptors such as SQSTM1 exist in the viral envelope [140]. Indeed, SQSTM1 appears to target precipitate tegument proteins or tegument protein complexes before the virion maturation completion [140]. Nevertheless, inhibition of autophagy still enhances replication of HCMV [139, 142, 143]. This indicates that despite autophagy being involved in the assembly of viral particles, it still plays an anti-viral function in the HCMV infection.

## Selective autophagy in viral infection

Virus-induced autophagy degradation was firstly recognized as virophagy, which effectively reduces the intracellular load of the virus, but other types of selective autophagy, which exert various effects on viruses, are also triggered (Table 1).

**Table 1:** Selective autophagy in virus infection

Selective autophagy (SA)	Virus name	Targeted cargos	SA receptors	SA associated factors	Interaction with the autophagy machinery	Effects	Refs.
Virophagy	VSV	–	-	TLR7	Glycoprotein G of VSV is detected by TLR7, and activates autophagy via the nutrient-sensing phosphoinositide 3-kinase-Akt signalling pathway	Autophagy restricts VSV replication	[146]
	RVFV	–	p62	TLR-7, TRAF6, MYD88	Drosophila TLR-7 recognizes RVFV, which activates antiviral autophagy through TRAF6 and MyD88	Autophagy restricts RVFV replication	[147]
	Orsay virus	Pathogen proteins	–	Ubiquitin	Orsay virus upregulates and enriches functional domains associated with ubiquitin-mediated proteolysis, including the ubiquitin ligase adapter F-box and MATH domain genes; on this basis, knockdown of an autophagy gene increases the pathogen load	Activation of autophagy reduces the pathogen load of Orsay virus	[148]
	SINV	SINV capsid protein	P62	–	p62 interacts with SINV capsid protein; knockdown of p62 blocks the targeting of viral capsid to autophagosomes	Autophagy protects against SINV infection and slows down virus-induced cell death	[149]
	HBV	viral core proteins	P62	Galectin-9	Galectin-9 restricts HBV replication via p62 -mediated selective autophagy of viral core proteins	Autophagy restricts HBV replication	[150]
	HSV-1	HSV-1 nucleocapsids and viral particles	–	FANCC	The nucleocapsid of HSV-1 is captured by autophagosomes during its transition through the cytoplasm, which is regulated by FANCC	FANCC mediated virophagy reduces the host’s susceptibility to HSV-1	[151]
	IAV	–	–	ATG16L1	Activation of non‐canonical autophagy causes LAP to control the microbial infection in vivo, inhibiting the fusion of IAV envelope with endosomes and facilitating the presentation of antigens on MHC II molecules	Non-canonical autophagy limits lethal infection by IAV	[152, 153]
	CCHFV	–	–	LC3	CCHFV causes a massive lipidation of LC3 in hepatocytes	The genetic alteration of autophagy does not affect CCHFV replication	[155]
Mitophagy	IAV	Mitochondria	–	Ripk2	Ripk2^−/−^ cells (accumulation of damaged mitochondria in cells) infected with IAV exhibit activated NLRP3 and increased levels of IL-18 and IL-1β	NOD-RIPK2 mediated mitophagy protects the host against virally triggered immunopathology	[173]
	IAV	Mitochondria	–	MAVs	M2 protein increases the formation of ROS-dependent MAVS aggregates, while antagonizing the autophagy to reduce the MAVS aggregates degradation	M2 protein inhibits mitophagy and promotes innate immunity	[174]
	IAV	Mitochondria	PB1-F2	MAVs, TUMF	PB1-F2 protein acts as an autophagy receptor and mediates the induction of complete mitophagy by simultaneously interacting with LC3B and TUFM, thereby increasing MAVS degradation	PB1-F2 protein promotes mitophagy, and weakens the production of type I IFN	[165, 179]
	IAV	Mitochondria	NBR1	RIG-I; MAVs	PB1 preferentially associates with NBR1 that recognizes ubiquitinated MAVS and delivers it to autophagosomes for degradation	PB1 facilitates H7N9 virus infection by inhibiting innate immune signalling	[180]
	HIV	Mitochondria	P62	NLRP3	HIV infection causes mitochondrial accumulation, thereby reducing the negative regulation of NLRP3	Inhibition of mitophagy up-regulates inflammation	[162, 163]
	Human herpesvirus 8 (HHV-8)	Mitochondria	NIX	–	HHV-8-encoded viral interferon regulatory factor 1 (vIRF-1) binds to NIX to activate mitophagy	Activation of mitophagy maintains mitochondrial homeostasis and promotes HHV-8 replication	[164]
	EBV	Mitochondria	NDP52	PINK1, MAVs-STING;	EBV encoded BHRF1 disturbs mitochondrial dynamics and stimulates mitophagy	Mitophagy inhibits the type I interferon response	[167]
	HPIV3	Mitochondria	–	TUFM	The M protein of HPIV3 translocates to mitochondria and interacts with TUFM to induce mitophagy	Mitophagy inhibits the type I interferon response	[168]
	SARS-CoV-2	Mitochondria	–	RIG-I-MAVs	The M protein of SARS-COV2 translocates to mitochondria to induce mitophagy	Mitophagy inhibits RIG-MAVS-mediated IFN-β signalling	[170]
ER-phagy	DENV/ ZIKV	ER	FAM134B	–	DENV/ ZIKV NS3-mediated cleavage of FAM134B blocks the formation of ER and viral protein-enriched autophagosomes	Flavivirus inhibits ER-phagy to promote its replication	[182]
	DENV/ ZIKV	ER	FAM134B	BPIFB3	Depletion of BPIFB3 enhances the FAM134B-mediated ER-phagy	ER-phagy facilitates Flavivirus replication	[184]
	EBOV	ER	FAM134B	–	FAM134B-mediated ER-phagy contributes to the degradation of viral components accumulated in the ER	Replication of EBOV is limited by FAM134B-dependent ER-phagy	[183]
	WNV/DENV/ ZIKV	ER	RTN3.1A	–	WNV encoded NS4A protein binds RTN3.1A to hijack ER membranes, generates a replication permissive niche to remodel the host membrane and stabilize the viral protein in the ER	Flaviviruses target RTN3.1A to avoid ER-phagy, thereby promoting viral replication	[186]
Lipophagy	DENV	Lipid droplets	–	–	Lipophagy is activated and reposited triglycerides are depleted, resulting in an increase in the release of β-oxidized fatty acids in mitochondria, which generates ATP	Lipophagy- generated ATP is required for virus replication and assembly	[191]
	DENV/ ZIKV	Lipid droplets	–	AUP1	DENV-NS4A exploits the acyltransferase activity of AUP1 to trigger lipophagy and leads AUP1 to relocalize from LDs to autophagosomes upon infection	Flaviviruses exploit the AUP1 to trigger lipophagy and increase progeny virus production	[192]
Aggrephagy	MCMV	Aggregates	–	RIPK1, NEMO, TBC1D, VPS26B 5	MCMV M45 protein requires IPAM to induce aggregation and degradation of RIPK1 and NEMO, then M45 recruits TBC1D5 and VPS26B to facilitate degradation of aggregates	MCMV induced-aggrephagy contributes to immune evasion of the virus and cell viability	[197]
Ferritinophagy	HCMV	Ferritin	NCOA4	–	pUL38 protein of HCMV antagonizes USP24 to reduce ferritinophagy	Reduced ferritinophagy increases cell viability and successful virus infection	[204]
	HPIV2	Ferritin	NCOA4	FTH1	V-2 protein of HPIV2 weakens ferritinophagy by interfering with the interaction between FTH1 and NCOA4	Reduced ferritinophagy allows infected cells to avoid apoptotic cell death, leading to effective viral replication of HPIV2	[205]

### Virophagy

Virophagy, also called xenophagy, is an important antiviral defense mechanism that not only targets the virus or viral protein for degradation but also promotes the host’s immune responses, such as inflammation regulation, antigen recognition and presentation. However, the molecular mechanism of autophagy recognizing whole virus particles or viral components and targeting them to autophagosomes has not been sufficiently investigated.

In the model organisms, *Drosophila* and *Caenorhabditis elegans*, virophagy is considered to be an inherent antiviral program [144, 145]. The lack of adaptive immune interference in these organisms provides unique conditions for studying the contribution of autophagy to innate immunity, especially epithelial defense. For instance, mutations in the autophagy genes, ATG18/WIPI2, ATG1/ULK1, ATG5, and ATG8A/LC3, in *D. melanogaster* S2 cells increase the susceptibility of *Drosophila* to vesicular stomatitis virus (VSV) [146]. Another study showed that during the Rift Valley Fever Virus (RVFV) infection, TLR7-mediated activation of autophagy limits RVFV replication and reduces mortality, while a knock-down of key autophagy components in *C. elegans* (e.g. ATG8/LGG-1 and SQSTM1/SQST-1) increased the load of the virus [147]. Correspondingly, autophagy is activated through starvation or through the autophagy negative regulator MTOR/LET-363, which reduces the pathogen load of Orsay virus [148]. These findings may provide evidence that the original function of autophagy is to eliminate and degrade harmful microorganisms that manage to enter the cytoplasm. However, it is surprising that in higher eukaryotes, the function of autophagy is gradually hijacked by viruses, which may be the result of the co-evolution between viruses and eukaryotes.

In other regards, virophagy prevents tissue injury and host cellular death by inhibiting the inflammatory cytokines production and intracellular microbes removal. Previous studies have shown that the capsid protein of Sindbis virus (SINV) is degraded through P62, and that ATG5 disruption in SINV-infected neurons decreases viral proteins clearance, and also results in the accumulation of cellular p62 and increased cell death [149]. In the same way, galectin-9 restricts hepatitis B virus (HBV) replication via p62-mediated selective autophagy of viral core proteins [150]. Genetic deletion of the Fanconi anemia (FA) pathway genes with DNA damage repair function blocks the virophagy and heightens susceptibility to lethal viral encephalitis during the SINV and HSV-1 infection [151].

The importance of non-canonical forms of virophagy in the host antiviral immune process has recently received extensive scientific attention. It was reported that the WD40 domain of ATG16L1 plays a critical role in the LC3 lipidation on single membranes during non-canonical autophagy [152]. Mice lacking the WD40 domain are extraordinarily sensitive to the low-pathogenicity IAV, and they suffer serious inflammatory pathological damage in the lungs; this is due to the non-canonical autophagy slowing the fusion of IAV envelopes with endosomes and down-regulating the IFN responsive genes [153]. In addition, non-canonical autophagy also facilitates the presentation of major histocompatibility complex class II (MHC II) antigens in IAV-infected mouse dendritic cells (DCs) [152]. When the autophagy levels are reduced, the beneficial enteric virus becomes pathogenic. It is probably because ATG16L1 in the epithelium prevents exacerbated TNFα, IFNγ and commensal bacteria-dependent intestinal injury after murine norovirus (MNV) infection [154]. In another study, massive amounts of lipidated LC3 were observed in ATG5, ATG7, or BECN1-silenced hepatocytes infected with Crimean Congo hemorrhagic fever virus (CCHFV). This implies the occurrence of non-canonical autophagy, but this accumulated lipidated LC3 seems to have no effect on virus replication [155]. Remarkably, a new alternative lipidation mechanism of ATG8-PS in the lysosomal compartment in the process of non-canonical autophagy was discovered recently; being different from the canonical conjugation of ATG8 protein to PE, ATG8-PS conjugation is a unique “molecular signature” for the non-canonical autophagy [156]. It has been confirmed that the influenza virus induces the non-canonical ATG8-PS autophagy, but it is still not clear how this unique modification affects virus replication [156].

### Mitophagy

Mitophagy is a vital form of autophagy that specifically degrades dysfunctional or redundant mitochondria. Since the accumulation of dysfunctional mitochondria induces a series of immune responses, mitophagy limits the secretion of inflammatory cytokines and directly regulates the presentation of mitochondrial antigens and immune cell homeostasis [157]. It is known that promoting mitophagy inhibits the secretion of type I IFN, which depends on the increased ROS production and mitochondrial retention [158, 159]. Inhibition of mitophagy activates Nod-like receptor protein 3 (NLRP3) inflammasome to further increase the secretion of IL-1β/IL-18 and the expression of NF-Κb [160]. Therefore, mitophagy is likely to be usurped by viruses for suppression of antiviral immunity, or be inhibited to cause mitochondrial degradation dysfunction, resulting in a strong immune response and severe damage to the host. HIV [161–163], herpesviruses [164], influenza viruses [165, 166], EBV [167], HPIV3 [168], senecavirus A [169] and SARS-CoV-2 [170–172] all appear to possess this ability.

Considering influenza viruses as an example: NOD2 Receptor interacting protein kinase 2 (Ripk2)^−/−^ cells exhibit accumulation of damaged mitochondria, but Ripk2^−/−^ cells are susceptible to IAV. After infection, IAV activates the NLRP3 and increases the levels of IL-18 and IL-1β. Therefore, NOD-RIPK2 signal transduction protects against virally triggered immunopathology by negatively regulating NLRP3 through mitophagy [173]. Our study confirmed that the IAV M2 protein increases the formation of ROS-dependent mitochondrial antiviral signalling protein (MAVS) aggregates [174]. It antagonizes autophagy and competes with ATG5 and LC3B to bind to MAVS, which reduces the formation of LC3B-MAVS and ATG5-MAVS complexes, as well as degradation of MAVS aggregates; followed by elevating the MAVS-mediated innate immune response [174]. Furthermore, the high molecular weight aggregates of the IAV virulence protein, PB1-F2, can be transferred to the inner membrane of mitochondria through the TOMM40 channel. This process reduces the membrane potential and promotes the fragmentation of mitochondria, which in turn promotes the activation of NLRP3 [175–178]. On the other hand, PB1-F2 protein acts as an autophagy receptor and mediates the induction of complete mitochondrial autophagy by simultaneously interacting with LC3B and the mitochondrial protein, the Tu elongation factor, mitochondrial (TUFM). This interaction increases MAVS degradation and weakens the production of type I IFN [165, 179]. A recent investigation showed that the PB1 protein of IAV also suppresses the innate immune response by targeting MAVS for NBR1-mediated selective autophagic degradation [180].

### ER-phagy or reticulophagy

The ER is a highly dynamic network that has a central role in cell metabolism and cellular organization. ER‐phagy contributes to the remodelling of the network under fluctuating conditions to ensure continuous normal functioning of ER and minimize stress [181]. As mentioned earlier, ER is the main membrane source of DMVs and viral replication or assembly site for viruses such as flaviviruses, CoVs and picornaviruses. Therefore, ER-phagy exerts innate antiviral functions against this group of viruses.

FAM134B is an important ER-phagy receptor, as its absence helps ER expansion and leads to ER stress. Various lines of evidence suggested that the replication of flavivirus and ebola virus (EBOV) are both limited by the FAM134B-dependent ER-phagy [182, 183]. However, flavivirus NS3-encoded protease and NS3 cofactor NS2B can cleave FAM134B to largely avoid this limitation [182]. Consistent with the above report, depletion of BPIFB3 improves the FAM134B ER-phagy and impairs the replication of flavivirus [184]. Another the ER-phagy receptor, RTN3, has been implicated in the remodelling of ER tubules in response to pathogen infections [185]. Flavivirus targeting RTN3.1A hijacks the ER-phagy by the NS4A protein of WNV to remodel the host membrane and stabilize the viral protein in the ER, but RTN3 interacts with NS4B of the HCV to abolish the NS4B self-interaction, thus negatively regulating viral replication [186, 187].

### Lipophagy

Autophagy also regulates lipid metabolism by modifying lipid droplets (LDs), a process termed lipophagy [188, 189]. LDs are composed of a neutral lipid core and surrounded by a monolayer of phospholipids. There are several proteins on the surface of LDs, which are used to supply energy when required by cells [190].

DENV induces autophagy to regulate lipid metabolism, which requires components of the autophagocytic machinery to achieve robust replication [191, 192]. During DENV or ZIKV infection, lipophagy is activated and stored triglycerides are depleted, which increases the release of β-oxidized fatty acids in mitochondria, thereby releasing the energy required for virus replication and assembly. The LDs then became a hotbed for viral replication [192–194]. Adding exogenous free fatty acids to autophagy-deficient cells restores the DENV replication. Furthermore, the application of Etomoxir, which blocks the transport of fatty acids to the mitochondria, blocks this process [191].

### Aggrephagy

Newly synthesized proteins need to be folded properly, but it is frequently hindered by oxidative stress, transcriptional/translational errors or mutations that cause protein misfolding [195]. Misfolded proteins form aggregates, which are then removed by aggrephagy. In the past, aggrephagy disorder was believed to be involved in the onset of many neurodegenerative diseases [196], it has been discovered that herpesviruses infections induce aggrephagy, which is a typical example of a conserved immune system evasion mechanism [197].

According to the latest reports, murine cytomegalovirus (MCMV) M45 protein motivates the aggregation and subsequent degradation of the receptor-interacting protein kinase 1 (RIPK1) and the NF-κB essential modulator (NEMO) [197]. The aggregation of RIPK1 and NEMO blocks antiviral responses such as the induction of necroptosis and the activation of NF-κB, and in that way contributes to the immune evasion of virus and cell viability. M45 requires an “induced protein aggregation motif (IPAM)” to induce the target proteins aggregation, then M45 recruits the LC3-interacting adaptor protein, TBC1D5 and VPS26B, facilitating degradation of aggregates [197]. Of note, some herpesviruses encode M45-homologous proteins containing the IPAM, such as EBV BORF2, HSV-1 ICP6, HSV-2 ICP10 and HHV-8 ORF61. Experimental results show that HSV-1 ICP6 has comparable activity to M45 [197].

### Ferritinophagy

Ferritinophagy is a special form of autophagy that specifically targets iron-sequestering protein ferritin for maintaining cellular iron homeostasis [189]. Although iron is an important part of various enzymes and proteins, excess free iron induces oxidative stress and the formation of ROS, which accelerates the cell death [198]. Ferritinophagy is regulated by the nuclear receptor coactivator 4 (NCOA4), which binds ferritin and marks it as autophagic cargo for iron recycling under low iron conditions [199]. At the same time, the replication of various viruses is affected by the iron concentration; these comprise HCV [200], HSV-1 [201], bovine viral diarrhea virus (BVDV) [201], HIV-1 [202], WNV [203], HCMV [204] and HPIV2 [205].

In some studies, inhibition of ferritinophagy has been recognised as a potential mechanism of prevention of cell death during viral infection. For example, the pUL38 protein of HCMV binds to USP24 to antagonize the cellular stress response, thereby preventing premature cell death [204]. During the HCMV infection, protein levels of NCOA4 and ferritinophagy are regulated, and Tiron and iron chelators ciclopirox olamine specifically protect cells from pUL38-deficient HCMV infection-induced cell death [204]. This shows that pUL38 antagonizes USP24 to reduce ferritinophagy and increase cell viability and successful virus infection. Similarly, the V-2 protein of HPIV2 weakens ferritinophagy by interfering with the interaction between the ferritin heavy chain 1 (FTH1) and NCOA4, allowing infected cells to avoid apoptotic cell death and facilitating effective viral replication of HPIV2 [205].

## Autophagy and the innate immunity in viral infections

### Antiviral interferon responses, inflammation and autophagy

**Fig. 4:**
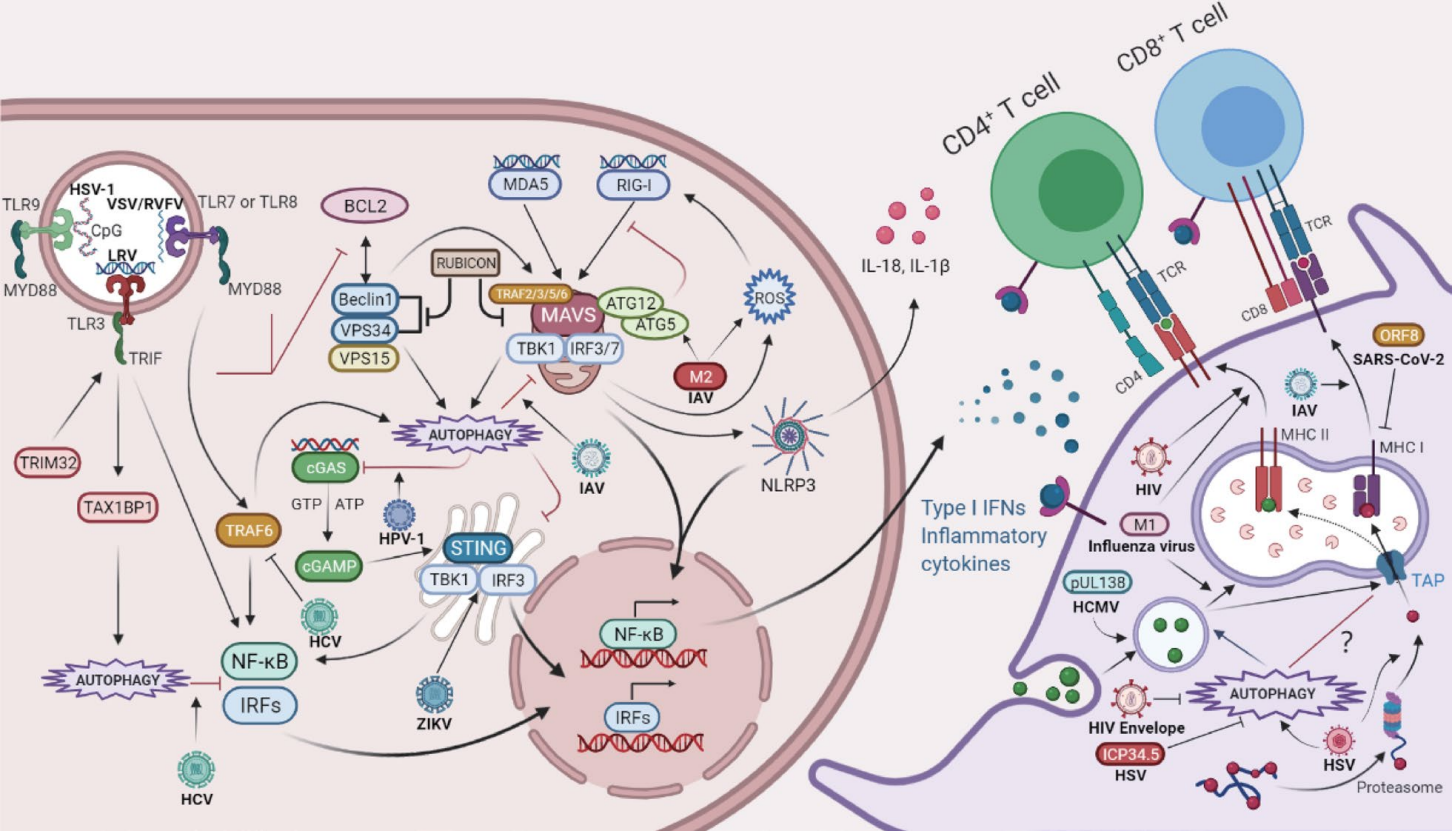
Autophagy and the innate immune in viral infection. The genetic material of RNA or DNA viruses is recognized by PRRs or cGAS, which facilitate viral induction of antiviral autophagy to improve the IFN production, thereby limiting virus replication. Specifically, VSV and RVFV activate antiviral autophagy and increase the production of type I IFNs through TLR-7 and MYD88 signal transduction; while LRV achieves it through TLR3 and TRIF, which triggers the degradation of NLRP3; HSV activates autophagy and induces interferon production through TLR9. STING-dependent autophagy induced by inflammation limits ZIKV infection. IAV M2 protein increases the formation of MAVS aggregates. It antagonizes autophagy through reducing the formation of ATG5-MAVS and LC3B-MAVS complexes, thereby enhancing the innate immune response. Conversely, HCV inhibits the innate immune response by inducing the autophagic degradation of TRAF6. During the HPV-1 infection, the interaction between cGAS and Beclin1 not only halts the production of IFN by inhibiting the synthesis of cGAMP, but also prevents excessive activation of cGAS to sustain systematic immune balance by enhancing autophagy degradation of viral DNAs. APCs initiate adaptive immunity by presenting protein fragments through MHC. M1 protein of Influenza is targeted by LC3 to autophagosomes, which fuse with MIIC to enhance the antigen presentation of CD4^+^ T cells. LC3 combined with HIV/SIV gag antigen targeted to autophagosomes enhance the HIV-specific CD4^+^ T cell response. HIV-1 envelope and ICP34.5 of HSV1 inhibit autophagy in DCs, escaping MHC-restricted presentation of its antigens. ORF8 of SARS-CoV-2 directly interacts with MHC Ι and mediates its down-regulation through autophagy to evade immune surveillance. HSV-1 infection induces autophagy and increases the presentation of peptides derived from HSV-1 glycoprotein B to CD8^+^ T cells in a manner that requires proteasome function and secretion pathways. Similarly, pUL138 of HCMV is presented by autophagy in a TAP-independent manner that involves MHC I loading in endosomal compartments

The viral invasion will trigger the activation of some specific PRRs, including: 1) Toll-like receptors (TLRs), such as TLR3 (dsRNA), TLR7 and TLR8 (ssRNA), and TLR9 (DNA with unmethylated CpG sites); 2) RIG-I like receptors (RLRs) (viral RNAs); and 3) Nod-like receptors (NLRs) [206]. Moreover, the cytosolic DNA sensor, cyclic GMP–AMP (cGAMP) synthase (cGAS), recognizes dsDNA during the DNA virus infection [206]. TLR7, TLR8 and TLR9 recruit the adaptor protein, myeloid differentiation primary response 88 (MYD88), while TLR3 recruits another type of adaptor, TIR-domain-containing adapter-inducing interferon-β (TRIF). Both adaptors activate NF-κB to synthesize inflammatory factors or the interferon pathway to induce IFN production in plasmacytoid pDC [207, 208]. MYD88 also recruits interleukin 1 receptor-associated kinase (IRAK) 1 and IRAK4 [209]. IRAK1 is phosphorylated to recruit E3 ubiquitin ligase and the scaffold protein, TNF receptor-associated factor 6 (TRAF6) [209]. Ubiquitinated TRAF6 induces the phosphorylation of the inhibitor of the IκB kinase (IKK) complex, activating the NF-κB [210]. Cytosolic viral DNA triggers STING1 through binding to cGAMP, resulting in the production of type I IFNs [211]. STING1 upregulates the expression of NF-κB-dependent pro-inflammatory cytokines [212]. Nevertheless, ATG9a inhibits the STING1 aggregation on Golgi apparatus-derived compartments to regulate the innate immune response; AMPK and ULK1 mediate the phosphorylation of STING1, which leads to the degradation of STING1, thereby limiting cytokine levels [213]. The RIG-I-MAVS-TRAF6 signal transduction axis is required for the RIG-I-mediated autophagy. After activation of RIG-I, Beclin1 translocates to mitochondria and then interacts with TRAF6 [214]. MAVS binds to TRAF2, TRAF3, TRAF5, or TRAF6 through its PRR domain, which promotes the activation of the TBK1 complex [215, 216]. The TBK1 complex promotes homodimerization and phosphorylation of interferon regulatory factors (IRFs) to activate IRFs, which then transfer to the nucleus where they link to IFN-stimulated response elements and motivate the transcription of target genes [216]. Moreover, TLR signal transduction enhances the interaction between TRIF or MyD88 and Beclin1, and reduces the binding of Beclin1 to BCL-2, which ultimately activates autophagy [217]. In contrast, tripartite motif-containing protein 32 (TRIM32) targets TRIF to negatively regulate TLR3-mediated immune responses for degradation of TAX1BP1-mediated selective autophagy [218]. Mitochondria exert antiviral functions through MAVS. After RIG-I recognizes the RNA produced by a viral infection and replication, it recruits MAVS to locate on the mitochondria and triggers MAVS activation. MAVS activation further activates IRFs and NF-κB, leading to the expression of IFN and pro-inflammatory cytokines [219]. The ATG5-ATG12 complex affects the formation and stability of MAVS aggregates by directly binding to the Caspase recruitment domain (CARD) of MAVS and RIG-I, thereby negatively regulating the signal transduction of the RLRs pathway [175, 220]. However, the absence of autophagy results in ROS-dependent signal transmission of RLRs [159]. Therefore, autophagy may be used as a negative feedback mechanism to regulate the type I IFN response. In parallel, autophagy removes mitochondria, leading to a reduced release of mitochondrial-derived damage-associated molecular patterns (DAMPs) and suppression of the NLRP3 inflammasome activation [221]. Rubicon is a protein that interacts with the Beclin1-VPS34 complex that inhibits the activity of CARD9, BCL10, and MALT1 (CBM complex) by binding to CARD9, thereby terminating RIG-I- or MDA5-mediated pro-inflammatory signal transduction [222] (Fig. 4).

Consequently, the relationship between autophagy and the immune response during viral infection is highly complicated and must be specifically analyzed according to different viral infections. Due to almost all viral infections inducing a complex immune response, below we provide descriptions of some representative viruses.

Specifically, autophagy-deficient ATG5 pDCs decrease TLR7-dependent IFNs production during the VSV and Sendai virus (SeV) infection [223]. Moreover, TLR-7 and MyD88 signal transduction hinders the RVFV replication in *Drosophila* and mammals by activating the antiviral autophagy [147]. Leishmania RNA virus (LRV) induces type I IFN production by activating TLR3 and TRIF, which triggers the ATG5-mediated autophagy-induced degradation of NLRP3 inflammasome in macrophages [224]. ATG5 also facilitates the production of TLR9-induced IFN-I in pDCs infected with HSV-1 [225]. STING1 is essential for an RNA-virus triggered autophagy, foot-and-mouth disease virus (FMDV)-induced integrated stress response originates from RIG-I, which transmits signals to STING1 and leads to degradation of STING1 itself [226]. In addition, STING-dependent autophagy induced by inflammation has been shown to limit ZIKV infection in the *Drosophila* brain [227, 228] (Fig. 4).

Conversely, HCV inhibits the host's innate immune response by inducing the autophagic degradation of TRAF6 [229]. Srikanta et al. found that HCV replication induces chronic ER stress in persistently infected cells and an autophagic response that selectively impaired the type I IFN signalling [230]. During HPV-1 infection, the interaction between cGAS and Beclin1 not only halts the production of IFN by inhibiting the synthesis of cGAMP, but also prevents excessive activation of cGAS to sustain systematic immune balance by enhancing autophagy-mediated degradation of cytosolic viral DNAs [231]. As mentioned in the Mitophagy part, the virus controls the RIG-I/MAVS-mediated production of IFN-I and activation of inflammasomes by promoting mitochondrial autophagy, which will not be reiterated. OTUD7B/Cezanne (OTU deubiquitinase 7B) acts as a negative regulator of antiviral immunity by deubiquitinates SQSTM1/p62 and promotes IRF3 degradation [232]. In addition, a newly discovered selective autophagy receptor CCDC50 targets RIG-I/MDA5 and degrades them after infection with VSV, SEV, and EMCV, thereby inhibiting IRF3/7 activation and NF-κB-mediated inflammation to enhance virus replication [41] (Fig. 4).

Collectively, the interaction between autophagy and the immune response is a double-edged sword in viral infection. On one hand, the activation of TLRs, RLRs, or cGAS-STING by viral infection may help to induce autophagy to improve the IFN production, thereby limiting virus replication; on the other hand, autophagy degrades damaged organelles and immune signal transduction proteins to impair the immune response process, or in extreme cases, prevent excessive immune responses to maintain the homeostasis of the intracellular environment, thereby, thus eventually promoting replication of the virus.

### Autophagy and viral antigen presentation

Autophagy proteins are also involved in different aspects of antigen presentation. Antigen-presenting cells (APCs) are capable of initiating adaptive immune response by presenting protein fragments through MHC molecules. MHC class I (MHC I) is expressed in nucleated cell types. Intracellular antigens are processed by the proteasome and transported to the ER through the transporter associated with TAP, which then binds to MHC I, and is typically presented to CD8^+^ T cells [233]. MHC II and related molecules are expressed by APCs or by other cells after being stimulated by IFN-γ. MHC II molecules mainly load extracellular antigens in the late endosomal MHC II inclusion compartment (MIIC), and also load a part of endogenous antigens via a variety of intracellular pathways [234, 235], which are presented to CD4^+^ T cells [233, 236]. It is important to note that an extra mechanism of loading exogenous antigens onto MHC I molecules occurs through a process called cross-presentation [237]. After autophagosome cargo is degraded by lysosomes, the antigen can be presented via the MHC II and promote the activation of CD4^+^ T cells [238]. In addition, autophagy mediates the internalization and degradation of MHC I molecules to limit the presentation of antigen [208]. In DCs deficient with autophagy-related genes, VPS34, ATG5, or ATG7, the surface expression of MHC I and induction of CD8^+^ T cell activation is increased [239, 240]. Recent research also showed that MHC I is targeted for degradation by the autophagy pathway involving the selective autophagy receptor NBR1 [241]. In contrast, some studies have provided evidence that autophagy enhances the MHC I antigen presentation [242]. For example, HeLa cells treated with the selective PI3K inhibitor, 3-methyladenine, display reduced autophagy-mediated degradation of defective ribosomal products (DRiPs), which is also accompanied by enhanced proteasome degradation and class I antigen presentation [238, 243] (Fig. 4).

Early studies found that influenza matrix protein 1 (M1) is targeted by ATG8/LC3 to autophagosomes, and then autophagosomes continuously fuse with MIIC to enhance the antigen presentation to CD4^+^ cells clones [244]. Interestingly, proteasome-dependent endogenous antigen processing, but not autophagy, contributes to the global influenza CD4 ( +) response [245]. In addition, the DCs lacking ATG16L1 WD 40 CTD infected with IAV exhibited a reduced MHC II antigen presentation. It suggests that non-canonical autophagy may complement the MHC II antigen presentation process [152]. Research on HIV showed the LC3 fusion protein combined with HIV/SIV gag antigen targeted to autophagosomes can effectively enhance the HIV-specific CD4 ( +) T cell response [246]. Nevertheless, HIV-1 envelope and ICP34.5 of HSV1 inhibit autophagy in DC and escape MHC-restricted presentation of its antigens [247] (Fig. 4).

The effect of autophagy on MHC I antigen presentation appears to be paradoxical, as there are differences in MCH I antigen presentation induced by specific viral infections. During the IAV and lymphocytic choriomeningitis virus (LCMV) infection, a lack of ATG5 leads to an enhanced virus-specific CD8^+^ T cell response [239]. DCs lacking VPS34 display enhanced presentation of chicken ovalbumin (OVA), IAV, and LCMV antigens to CD8^+^ T cells [240]. Remarkably, a recent study confirmed that an open reading frame 8 (ORF8) of SARS-CoV-2 directly interacts with MHC Ι and mediates its down-regulation through Beclin1–mediated selective autophagy to evade immune surveillance [248]. However, after HSV-1 infects macrophages to induce autophagy, it increases the presentation of a peptide derived from the HSV-1 glycoprotein B to CD8^+^ T cells in a manner that requires proteasome function and secretion pathways [249]. Similarly, an HCMV-encoded antigen of the type I integral membrane protein, pUL138, can be presented by autophagy in a TAP-independent manner that involves MHC I loading in endosomal compartments [242] (Fig. 4).

In conclusion, autophagosomes induced by viral infection carry viral components and fuse with MIIC to provide proteins for MHC II presentation to CD4^+^ cells to induce an antiviral immune response, but some viruses escape this immune process by reducing autophagy. Viral proteins and autophagy proteins mediate the direct degradation of MHC I, and autophagy deficiency leads to a virus-specific CD8^+^ T cell response enhancement. However, it seems that autophagy does not affect other ways of MHC I antigen presentation, which requires further in-depth research.

## Virus-specific induction of autophagy

Recently, Dr. Beth Levine’s laboratory utilized genome-wide siRNA screening to discover a type of virus-induced autophagy mediated by sorting nexin 5 (SNX5), which has subsequently attracted widespread attention [250]. Virus-induced autophagy differs from the general autophagy mediated by starvation or mTOR, and the non-canonical forms of autophagy induced by bacteria or osmotic stress. Both SNX5-deficient cells and SNX5-knockout mice are more susceptible to SIN, HSV-1, WNV, CHIKV, and other viruses, but there is no difference in the susceptibility to recombinant viruses that have the ability to inhibit autophagy [250]. When the virus enters the endosome, SNX5 increases the curvature of the membrane through the BAR domain to activate the autophagy-related PI3KC3-C1 kinase complex, and generates the key autophagy initiation signal, PI (3) P, on the endosome membrane, thus activating the autophagy [250]. However, the mechanism by which luminal viruses stimulate the SNX5-PI3KC3 axis on the cytoplasmic surface of endosomes is still unidentified. These findings confirm the existence of SNX5-mediated activation of the viral autophagy signalling pathway, which represents a novel and important host defense mechanism.

Indeed, the comparative characterization of the SINV proteome from mammalian and invertebrate hosts identified SNX5 as an important host factor for alphavirus replication [251]. Asuka et al. have previously reported co-localization of fluorescently-labeled EBOV particles with SNX5 in the process of researching the internalization mechanism of EBOV [252]. In addition, SNX5 and PI (3) P play a key role in the formation of the viral replicase complexes (VRCs) bound on the organelle membrane of the tomato bushy stunt virus (TBSV) [253]. Importantly, HCMV-encoded UL35 binds to and negatively regulates SNX5, thereby regulating cellular transport pathways that affect the virus assembly process [254]. These results indicate that the endosomal membrane remodelling process affects the entry, replication, and assembly processes of many viruses. The regulatory relationships among SNX5, various viruses and autophagy require further research.

## Conclusion

As a ubiquitous metabolic pathway in most multicellular organisms, autophagy exhibits strong defense capabilities against viral invasion, including the regulation of inflammation, promotion of antigen presentation, and the degradation of viral components or particles. Nevertheless, the diversity of methods exploited by different viruses to manipulate the autophagy pathway is equally impressive. Viruses can use the autophagy pathway to interfere with the immune response or prevent cell death, and to take advantage of autophagy-related metabolites. Some viruses can even directly exploit autophagosomes for assembly, or use secretory autophagy to promote the egress of virus particles and cell–cell spreading, and avoid antibody neutralization. However, whether autophagy contributes to or inhibits viral replication is indeterminate and dependent on several factors, including types of infected cells, the virus strains, and conditions of infection. Another key point that should not be ignored is that most of experimental designs investigating virus role in autophagy are carried out in cancer cell lines, given that autophagy plays a great regulatory role in cancer cell survival mechanisms, the effect of autophagy on virus replication may require in vivo experiments to be more convincing. In addition, viruses that cannot be assembled inside the cell (e.g. HPIV3, IAV and other viruses), can induce the accumulation of autophagosomes, and hinder the membrane fusion between autophagosomes and lysosomes. The specific role of these accumulated autophagosomes requires further research. Importantly, some new discoveries, such as the influence of ATG8-PS alternative lipidation mechanism on virus replication in viruses-triggered non-classical autophagy and mechanisms by which viruses specifically induce autophagy, offer novel directions for future research. This review provides an important foundation for the development of broad-spectrum antiviral treatment strategies and drugs based on the regulation of autophagy.

## Data Availability

All data relevant to this review are included in the text, references, table, and figures.

## Abbreviations

APC: Antigen-presenting cell
ATG: Autophagy-related
Bcl-2: B cell lymphoma-2
BVDV: Bovine viral diarrhea virus
CARD: Caspase recruitment domain
CCHFV: Crimean-Congo hemorrhagic fever virus
CHIKV: Chikungunya virus
CoV: Coronavirus
CVB3: Coxsackievirus B3
CVB4: Coxsackievirus B4
DENV: Dengue virus
DMV: Double membranous vesicle
EBOV: Ebolavirus
EBV: Epstein-Barr virus
EMCV: Encephalomyocarditis virus
ER: Endoplasmic reticulum
ESCRT: Endosomal sorting complex required for transport
EV71: Enterovirus 71
EV-D68: Enterovirus 68
FMDV: Foot-and-mouth disease virus
HCMV: Human Cytomegalovirus
HCoV-NL63: Human Coronavirus NL63
HCV: Hepatitis C virus
HIV: Human immunodeficiency virus
HPIV3: Human parainfluenza virus type 3
HRV: Human rhinovirus
HSV-1: Herpes simplex virus types 1
IAV: Influenza A virus
IFN: Interferon
IM: Isolation membrane
IRAK: Interleukin 1 receptor associated kinase
IRF: Interferon regulatory factors
IRF: Interferon regulatory factors
K63: Lys63
KSHV: Kaposi’s sarcoma-associated herpesvirus
LC3: Ligase of microtubule-associated protein L chain 3
LCMV: Lymphocytic choriomeningitis virus
LD: Lipid droplets
LRV: Leishmania RNA virus
MAVS: Mitochondrial antiviral signaling protein
MCMV: Murid herpesvirus 1
MERS-CoV: Middle East Respiratory Syndrome Coronavirus
MHC: Major histocompatibility complex
MHV: Mouse Hepatitis Virus
MVB: Multivesicular bodies
MyD88: Myeloiddifferentiationfactor88
NF-κB: Nuclear factor-κB
NLRP3: Nod-like receptor protein 3
PAMPs: Pathogen-associated molecular patterns
PI (3) P: Phosphatidylinositol 3-phosphate
PRRs: Pattern-recognition receptors
PV: Poliovirus
RIG-I: Retinoic acid-inducible gene I
RLR: RIG-I-like receptor
ROS: Reactive oxygen species
RVFV: Rift Valley Fever Virus
SARS-CoV: Severe Acute Respiratory Syndrome Coronavirus
SINV: Sindbis virus
SNARE: Soluble N-ethylmaleimide-sensitive factor attachment protein receptor
TAP: Transporter associated with antigen processing
TAX1BP1: TAX1-binding protein 1
TBK1: TANK binding kinase 1
TBSV: Tomato bushy stunt virus
TLRs: Toll-like receptors
TRAF: TNF receptor associated factor
TRIF: TIR domain-containing adaptor molecule 1
TRIM32: Motif-containing protein 32
VSV: Vesicular stomatitis virus
WNV: West Nile virus
ZIKV: Zika virus
CMA: Chaperone-mediated autophagy
ERGIC: ER-Golgi intermediate compartment
FTH1: Ferritin heavy chain 1
IRGM: Immunity-associated GTPase family M
LAP: LC3‐associated phagocytosis
MTOR: Mechanistic target of rapamycin
NBR1: Neighbor of BRCA1 gene
NCOA4: Nuclear receptor coactivator 4
PS: Phosphatidylserine
RTA: Replication and transcriptional activator
SFTSV: Severe fever with thrombocytopenia syndrome virus
SNX5: Sorting nexin 5
TGN: Trans-Golgi network
TSC: Tuberous sclerosis complex
v-GPCR: V-G protein-coupled receptor
